# Resistance of Arctic phytoplankton to ocean acidification and enhanced irradiance

**DOI:** 10.1007/s00300-017-2186-0

**Published:** 2017-08-09

**Authors:** C. J. M. Hoppe, N. Schuback, D. Semeniuk, K. Giesbrecht, J. Mol, H. Thomas, M. T. Maldonado, B. Rost, D. E. Varela, P. D. Tortell

**Affiliations:** 1 Department of Earth, Ocean and Atmospheric Sciences, University of British Columbia, Vancouver, BC Canada; 2 Marine Biogeosciences, Alfred Wegener Institute – Helmholtz Centre for Polar and Marine Research, Am Handelshafen 12, 27570 Bremerhaven, Germany; 3 Department of Physics and Astronomy, Curtin University, Perth, WA Australia; 4 School of Earth and Ocean Sciences, University of Victoria, Victoria, BC Canada; 5 Department of Oceanography, Dalhousie University, Halifax, NS Canada; 6 Department of Biology, University of Victoria, Victoria, BC Canada; 7 Department of Botany, University of British Columbia, Victoria, BC Canada; 8 Peter Wall Institute for Advanced Studies, University of British Columbia, Vancouver, Canada

**Keywords:** Multiple stressors, Climate change, Arctic Ocean, Diatoms, Primary production, Ocean acidification, Irradiances

## Abstract

The Arctic Ocean is a region particularly prone to ongoing ocean acidification (OA) and climate-driven changes. The influence of these changes on Arctic phytoplankton assemblages, however, remains poorly understood. In order to understand how OA and enhanced irradiances (e.g., resulting from sea–ice retreat) will alter the species composition, primary production, and eco-physiology of Arctic phytoplankton, we conducted an incubation experiment with an assemblage from Baffin Bay (71°N, 68°W) under different carbonate chemistry and irradiance regimes. Seawater was collected from just below the deep Chl *a* maximum, and the resident phytoplankton were exposed to 380 and 1000 µatm pCO_2_ at both 15 and 35% incident irradiance. On-deck incubations, in which temperatures were 6 °C above in situ conditions, were monitored for phytoplankton growth, biomass stoichiometry, net primary production, photo-physiology, and taxonomic composition. During the 8-day experiment, taxonomic diversity decreased and the diatom *Chaetoceros socialis* became increasingly dominant irrespective of light or CO_2_ levels. We found no statistically significant effects from either higher CO_2_ or light on physiological properties of phytoplankton during the experiment. We did, however, observe an initial 2-day stress response in all treatments, and slight photo-physiological responses to higher CO_2_ and light during the first five days of the incubation. Our results thus indicate high resistance of Arctic phytoplankton to OA and enhanced irradiance levels, challenging the commonly predicted stimulatory effects of enhanced CO_2_ and light availability for primary production.

**Electronic supplementary material:**

The online version of this article (doi:10.1007/s00300-017-2186-0) contains supplementary material, which is available to authorized users.

## Introduction

Ongoing global climate change has led to profound alterations in the marine environment, which are predicted to be particularly severe in the Arctic (Arctic Council 2013; Pörtner et al. 2014). The Arctic sea–ice cover is declining rapidly, with reductions in the summer minimum extent by more than 50%, a decline in mean thickness by over 60%, and a 30–60% decrease in snow on ice thickness since the 1970s (Stroeve et al. 2012; Webster et al. 2014; Lindsay and Schweiger 2015). These changes in sea–ice allow for more light penetration and longer growing seasons (Wassmann and Reigstad 2011; Nicolaus et al. 2012), potentially increasing Arctic Ocean primary production (Arrigo et al. 2008). Enhanced thermal stratification and freshening due to sea–ice melt and increasing river discharge may alter the light regime in the shallower upper mixed layer (Peterson et al. 2002; Steinacher et al. 2010). Such potential stimulatory effects, however, could be offset by reduced nutrient input from deeper waters (Wassmann and Reigstad 2011; Tremblay et al. 2015) or by higher UV radiation due to ozone depletion in the Arctic (Rex et al. 2004). Moreover, cold temperatures and low seawater alkalinity in some areas of the Arctic Ocean make the system particularly sensitive to anthropogenic CO_2_ loading. As a result, the effects of increasing CO_2_ concentrations and decreasing seawater pH (i.e., ocean acidification, OA) are especially pronounced in the Arctic Ocean (AMAP 2013; Tynan et al. 2015). Regions of particularly rapid change are the outflow shelves of the Arctic Ocean, including Baffin Bay. Over the past two decades, rapid sea–ice loss as well as warming, freshening, and associated changes in productivity patterns have been documented in these near-shore Arctic regions (Straneo and Heimbach 2013; Bergeron and Tremblay 2014; Soltwedel et al. 2015).

Interactive effects of different environmental drivers (Gao et al. 2012a) are thought to inevitably influence phytoplankton assemblages in the Arctic Ocean. In this region, future trends in primary production are critically dependent on the relative importance of different environmental drivers: beneficial effects of increased irradiances and potentially detrimental effects of decreased nutrient input, provided that the effects of enhanced stratification dominate over those of increased wind-driven mixing (Arrigo and van Dijken 2011; Vancoppenolle et al. 2013; Ardyna et al. 2014; Tremblay et al. 2015). Observational data are indispensable to estimate the potential effects of climate change on Arctic phytoplankton assemblages. However, even on decadal timescales, it is challenging to disentangle natural variability from anthropologically induced changes in the environment (Soltwedel et al. 2015). Experimental assessments of the various cause-effect relationships regarding single and interactive environmental drivers are thus needed to evaluate the consequences of climate change for ecosystem structure and functioning in the Arctic Ocean.

To the best of our knowledge, there have been only few experimental studies on Arctic phytoplankton assemblages directly assessing the effects of OA, i.e., potentially negative effects of decreased pH as well as positive effects of elevated CO_2_ concentrations (AMAP 2013). Results from recent field studies have shown a potential for positive OA-responses in primary production and phytoplankton growth (Engel et al. 2013; Coello-Camba et al. 2014; Holding et al. 2015). Increasing temperatures, however, seem to reduce potential benefits of increased CO_2_ concentrations (Holding et al. 2015), which could be due to a shift in CO_2_ optima to higher levels (Sett et al. 2014). Beyond changes in phytoplankton productivity, OA has been shown to influence Arctic phytoplankton community structure—i.e., the relative abundance of various taxonomic and functional groups (Newbold et al. 2012; Brussaard et al. 2013). Such OA-dependent changes in Arctic phytoplankton species abundances could cause substantial changes in ecosystem dynamics and their impact on biogeochemical cycles, yet the extent to which these few results can be extrapolated is currently uncertain.

While some studies have begun to examine the interactive effects of warming and OA in the Arctic (Coello-Camba et al. 2014; Holding et al. 2015; Pančić et al. 2015), little is known about the interactive effects of OA and increasing light levels in the Arctic (Hoppe Clara et al. 2017). Given the expected changes in both light availability and carbonate chemistry over the coming decades, it is important to investigate their interactive effects on Arctic primary producers. To do so, we conducted an incubation experiment with a phytoplankton assemblage from the Baffin Bay region. We simulated an upwelling event that would bring phytoplankton and nutrients from the deep Chl *a* maximum (DCM) to the surface, a phenomenon recently acknowledged to increase annual primary production in the Arctic due to the development of fall blooms (Ardyna et al. 2014). The phytoplankton assemblage was exposed to two pCO_2_ and two light levels in all possible combinations in on-deck incubations, and the effects on eco-physiology were monitored in order to investigate both isolated and interactive effects of OA and enhanced light.

## Materials and methods

### Experimental set-up

Our field work was conducted during the Arctic-GEOTRACES 2015 summer campaign on board the *CCGS Amundsen*. The experiment was initiated on August 6, 2015 in Baffin Bay near Clyde River (71°24.327′N, 68°36.057′W). At the sampling location, a depth profile of conductivity and temperature (CTD, seabird SBE9+), chlorophyll *a* fluorescence (Chl *a*; Seapoint SCF fluorometer) and photosynthetically active radiation (PAR, 400–700 nm; PNF-300 Profiling Natural Fluorometer, Biospherical Instruments) was measured. The mixed layer depth (MLD) was calculated from temperature and salinity-derived density profiles, using a density difference criterion relative to the surface (Δ*σ* = 0.125 kg m^−3^) (Levitus 1982).

We sampled phytoplankton from just below the DCM (45 m depth; Online Resource 1) using a trace metal clean rosette system modified according to Measures et al. (2008) and equipped with 12-L Teflon-coated GO-FLOs (General Oceanics, FL USA). Seawater from the GO-FLO bottles was dispensed into acid-cleaned 50-L carboys under HEPA-filtered air. Subsequently, seawater was pre-screened through acid-cleaned 100-µm nylon mesh to exclude grazers such as copepod nauplii. Please note that some larger phytoplankton cells or chains may have been excluded by this method, even though the assemblages were nonetheless dominated by chain-forming diatoms. Then, seawater was transferred into acid-clean 8-L polycarbonate bottles with custom-built fittings for aeration and sub-sampling.

The incubation bottles were kept in on-deck incubators, which were temperature-controlled using surface water collected by the ship’s underway system. Bottles were continuously bubbled with air of two defined pCO_2_ levels delivered through airstones from commercially prepared mixtures (380 and 1000 µatm), representing lower ambient (LC) and higher future atmospheric partial pressures (HC). The phytoplankton assemblages were exposed to these pCO_2_ levels at two light levels, i.e., 15% (LL, low light) and 35% (HL, high light) of incident PAR. The light quantity and spectral quality were chosen to represent contrasting irradiance regimes resulting from enhanced surface stratification, and were achieved by applying neutral density photographic film in combination with blue film (maximum transmission at approx. 460 nm; neutral density #209 and CT blue #202, Lee filters). Light levels in the incubator tanks were measured with a LI-1400 data logger (LI-COR) equipped with a 4π-sensor (Walz). Incident PAR was recorded continuously using a LI-1000 data logger and a LI-190SA cosine sensor (Li-COR) mounted on deck near the incubators. All treatments were conducted in triplicate, resulting in a total of 12 bottles for the whole experiment. Sea surface temperature was continuously logged in the inlet of the ship’s underway system.

To prevent nutrient limitation, nitrate, phosphate, and silicic acid from chelexed stock solutions were added in ratios appropriate for the region (Varela et al. 2013), yielding final concentrations of approx. 20 µmol L^−1^ NO_3_^−^, 2.5 µmol L^−1^ PO_4_^3−^, and 30 µmol L^−1^ Si(OH)_4_. After 5 days, incubations were diluted 20-fold yielding similar Chl *a* concentrations as during the start of the experiment with filtered seawater from the initial sampling location. The dilution step was used to allow the phytoplankton assemblages to acclimate to the experimental conditions, and for shifts in the species composition to occur, while also preventing nutrient limitation and/or drifts in carbonate chemistry. Water for the dilution of the experimental bottles was collected using a trace metal clean Teflon peristaltic pump and tubing system suspended with Kevlar line (Taylor et al. 2013). Dilution water was filtered through rinsed 0.2-µm filtration cartridges (AcroPak, Pall Corporation) and stored in the dark at 4 °C in 50-L carboys until use.

Phytoplankton growth in the incubation bottles was monitored by daily measurements of macronutrient concentrations, pH, basal, and maximal Chl *a* fluorescence yields (see below for details). The initial phytoplankton community as well as those present directly before the dilution and during the final sampling were assessed by sampling for taxonomy, by measuring a range of bulk stoichiometric parameters (ratios of Chl *a*, PON and bSi to POC), and by performing several physiological assays, as described below.

### Carbonate chemistry

Samples for the determination of dissolved inorganic carbon (DIC) and total alkalinity (TA) were collected in 250-mL borosilicate glass bottles with gas-tight stoppers. Samples for initial values were taken from the 50-L carboys during filling of the experimental bottles, while samples before dilutions and at the final sampling were taken directly from the experimental bottles. Samples were analyzed within 6 h after collection to minimize alteration by biological activity. DIC and TA were analyzed on board by coulometric and potentiometric titration, respectively, using a VINDTA 3C (Marianda) following the methods described in Dickson et al. (2007). Routine analyses of certified reference materials provided by A.G. Dickson (Scripps Institute of Oceanography) ensured that the uncertainty of DIC and TA measurements was smaller than 2 and 3 µmol kg^−1^, respectively.

Seawater pH on the total scale (pH_total_) was determined potentiometrically with a two-point calibrated glass reference electrode (IOline, Schott Instruments). A TRIS-based reference standard (Dickson et al. 2007) was used to convert from NBS to total scale and to correct for variability in electrode performance (reproducibility 0.02 units; *n* = 16). Seawater carbonate chemistry was calculated from TA and DIC using CO_2SYS_ (Pierrot et al. 2006) and the refitted dissociation constants of carbonic acid of Mehrbach et al. (Mehrbach et al. 1973; Millero et al. 2002). Dissociation constants for KHSO_4_ were taken from Dickson (Dickson 1990). pH and pCO_2_ levels are given for average in situ temperatures in the incubators during sampling, or as measured by the CTD in case of the initial sampling conditions (see above).

### Biomass composition

Samples for determination of total Chl *a* were gently filtered onto pre-combusted glass-fiber filters (GF/F, Whatman; <200 mmHg) every second morning together with the samples for photo-physiological measurements. Filters were kept out of direct light during sampling and filtrations, and were immediately placed into liquid nitrogen and stored at −20 °C until analysis. Chl *a* was extracted overnight at −20 °C in 8 mL 90% acetone. After removal of the filter, Chl *a* concentrations were determined on a fluorometer (Turner Designs), using an acidification step (1 M HCl) to determine phaeopigments (Knap et al. 1996). Size-fractionated Chl *a* was estimated by filtering samples onto stacked 5.0- and 0.6-μm polycarbonate filters (*Sterlitech* Corporation) separated by nylon drain disks (Millipore) as described in Semeniuk et al. (2009).

Particulate organic carbon (POC) and nitrogen (PON) were sampled at the initial and final time points as well as directly before the dilution by gentle filtration onto pre-combusted GF/F filters. Filters were stored in pre-combusted glass petri dishes at −20 °C. Filters were acid fumed over concentrated HCl for 2–3 days. Subsequently, filters were dried at 60 °C over night. Carbon and nitrogen contents were measured with a precision of ±1.3% on a CHN analyzer (vario MICRO cube, Elementar Americas) with a furnace temperature of 1050 °C and a flash combustion temperature of 1080 °C.

At the same time points, samples for the determination of biogenic silica (bSi [bSiO_2_]) were gently filtered onto 0.6-µm cellulose acetate filter (*Sterlitech* Corporation), and stored in plastic petri dishes at −20 °C until analysis. Subsequently, filters were dried over night at 60 °C. Alkaline hydrolysis with sodium hydroxide (NaOH) (Brzezinski and Nelson 1989) was used to convert bSi into Si(OH)_4_. The concentration of Si(OH)_4_ was measured based on the formation of beta silicomolybdic acid with a UV–Vis spectrophotometer (Beckman DU 530), using a reverse-order reagent blank (Brzezinski and Nelson 1986).

### Phytoplankton species composition

Samples for cell counts at the initial, dilution, and final time points were fixed with a combination of buffered-formalin (2% final concentration) and glutaraldehyde (0.1% final concentration) (J. Wiktor, pers. comm.). Samples were analyzed on a light microscope (Axiovert, Zeiss) after 24-h sedimentation in 10-mL Utermöhl chambers (Hydro-Bios). Unfortunately, sample aggregation due to strong precipitation of paraformaldehyde or trioxymethylene made quantitative cell counts impossible. We thus report only qualitative observations of large dominant groups.

Size distribution of the <10 µm fraction of the plankton communities was investigated by using flow cytometry (Marie et al. 2014). Duplicate samples were preserved by adding 3.5 mL of sample to 100 µL fixation solution (0.5% formaldehyde and 0.3% hexamine, final concentration). After gentle mixing, samples were stored at room temperature in the dark for 10 min, and subsequently frozen in liquid nitrogen and stored at −80 °C until analysis. Before analysis, samples were thawed on ice and mixed thoroughly. Analysis was performed based on red (FL3 channel, >670 nm) and green (FL1 channel, 533 ± 30 nm) fluorescence, as well as sideward and forward light scattering using a BD Accuri C6 flow cytometer equipped with a blue solid-state laser (488 nm excitation wavelength). Phytoplankton samples were primarily analyzed based on the cell’s auto-fluorescence signal (FL3; threshold = 800) on medium fluidics settings (35 µL min^−1^; 16 µm core size) with a limit of 50,000 events or 500 µL. Cell sizes (and some taxonomic information, derived from size and fluorescence characteristics) were based on previous measurements of calibration beads and phytoplankton cultures (I. Luddington, pers. comm.).

### Primary production and Si uptake assays

Net Primary production (NPP) was determined at the initial sampling time point, as well as on dilution and final days using 24-h incubations under the experimental light conditions (i.e., under LL and HL in on-deck incubators). An 18 µCi spike of NaH^14^CO_3_ (PerkinElmer, 53.1 mCi mmol^−1^ or 2.109 MBq mol^−1^ stock) was added to 180-mL subsamples. These subsamples were divided into duplicate 60-mL samples for incubations, while a 50-mL sample was immediately filtered after spiking (*T*_0_). Three 0.5 ml aliquots, added to 0.5 mL 1 M NaOH, were used to determine the total activity in each spiked sample (total counts, TC). After 24 h of incubation, samples were filtered onto GF/F filters, acidified with 0.5 mL 1 M HCl, and left to degas under a fumehood overnight. After addition of 10 mL of scintillation cocktail (ECOLUM™, MP Biomedicals), samples were vortexed and left to stand in the dark for approximately 12 h before counting on a liquid scintillation counter (DPM_sample_; Tri-Carb, PerkinElmer), using automatic quench correction and a counting time of 5 min. NPP rates [µg C (µg Chl *a*)^−1^ d^−1^] were calculated as

1 $${\text{NPP}} = \left( {\left[ {\text{DIC}} \right] \times \left( {{\text{DPM}}_{\text{sample}} {-}{\text{DPM}}_{T0} } \right) \times 1.05} \right)/\left( {{\text{DPM}}_{\text{TC}} \times \, t \, \times \, \left[ {{\text{Chl}}\,a} \right]} \right),$$

where [DIC] and [Chl *a*] denote the concentrations of DIC and Chl *a* in the sample, respectively. DPM_sample_ denotes the disintegrations per min (DPM) in each sample, DPM_T0_ reflects the *T*_0_ value, DPM_TC_ denotes the DPM of the TC sample, and t is the duration of the incubation. The value of 1.05 is used to correct for fractionation against ^14^C relative to ^12^C (Nielsen 1955).

Rates of biogenic silica production were measured at the end of the experiment from 24-h incubations using the radioisotope ^32^Si. For each sample, an acid-clean 250-mL polycarbonate bottle was filled to the neck (~300 mL) and spiked with 333 Bq (i.e., 0.009 µCi) of high specific activity ^32^Si (28,800 MBq or 778 mCi µmol^−1^ Si). Samples were incubated in on-deck incubators under the respective LL and HL conditions. Following incubation, samples were gently filtered using a vacuum pump (<200 mmHg) onto 0.6-µm polycarbonate filters. Filters were rinsed with 0.6-µm filtered seawater to remove excess tracer not incorporated into the particles and then placed on nylon disks to dry at room temperature. Once dry, samples were covered with Mylar film and stored until secular equilibrium was reached. The activity of ^32^Si in the samples was determined by gas-flow proportional counting at secular equilibrium using a Risø 25-5 low-level beta GM multicounter (Krause et al. 2011). Biogenic silica production rates (µmol Si L^−1^ d^−1^) were calculated as in Brzezinski and Phillips (1997).

### Photo-physiology assays and growth rates

Subsamples for photo-physiological measurements via fast repetition rate fluorometry (FRRF) were taken daily at approximately 1 h after local sunrise. Samples were kept at low light (<10 µmol photons m^−2^ s^−1^) and at incubator temperatures for at least 30 min before measurements, in order to achieve a dark-regulated photo-physiological state (i.e., all photochemical and non-photochemical quenching processes relaxed). All FRRF measurements were conducted on a benchtop FRRF instrument (Soliense Instuments) as described in Schuback et al. (2017). For each sample, a single turnover protocol (70 flashlets with 0.7 µs length and 2.5 µs interval, 87,800 µmol photons m^−2^ s^−1^ peak power intensity, resulting in a excitation sequence of 225 µs, providing ~7–12 photons per RCII) was applied to derive basal and maximal Chl *a* fluorescence (Chl F) yields *F*_o_ and *F*_m_, respectively. These were used to estimate *F*_v_/*F*_m_ (calculated as (*F*_m_ − *F*_o_)/*F*_m_), a measure of the quantum efficiency of charge separation in PSII. Furthermore, we derived the functional absorption cross section *σ*_PSII_ (Å^2^ RCII^−1^).

We observed a strong correlation between Chl *a* concentration in extracts and *F*_m_ (*r* ^2^ = 0.93, *n* = 92), and thus used the increase in *F*_m_ over time to calculate community biomass accumulation rate constants (*µ*) as an estimate for net growth rates. *µ* was calculated by exponentially fitting the daily *F*_m_ values of each bottle as a function of time between initiation and dilution as well as those after the dilution until the end of the experiment (see also Online Resource 2).

On dilution and final sampling days, we conducted additional measurements of steady-state light curves, as described in Schuback et al. (2017). Rates of initial charge separation in reaction center II (ETR_RCII_, mol e^−^ mol RCII s^−1^) were calculated from Chl F yields measured at nine incrementally increasing background PAR levels. The exponential model of Webb et al. (1974) was used to fit ETR_RCII_ versus light curves and derive the maximum, light-saturated rate $P_{ \hbox{max} }^{\text{RCII}}$ (mol e^−^ mol RCII^−1^ s^−1^), the light-depended increase in ETR_RCII_ before light saturation *α* ^RCII^ (mol e^−^ mol RCII^−1^ s^−1^ [µmol photons m^−2^ s^−1^]^−1^), and the light saturation parameter *E*_k_ (µmol photons m^−2^ s^−1^).

Two hour ^14^C-uptake light-response curves were conducted from all bottles on dilution and final days. A volume of 250 mL from each incubation bottle was spiked with NaH^14^CO_3_ (PerkinElmer, 53.1 mCi mmol^−1^ or 2.109 MBq mol^−1^ stock, final concentration 0.6 µCi mL^−1^), and the spiked sample was aliquoted into ten 20-mL glass scintillation vials, which were incubated for 2 h at ten light intensities ranging from 10 to 500 µmol photons m^−2^ s^−1^. Two 20-mL samples per bottle were filtered immediately after spiking (*T*_0_), and three 0.5 mL aliquots were taken from each spiked sample and added to 0.5 mL 1 N NaOH to determine the total activity (total counts, TC). After 2 h of incubation, samples were filtered onto 25-mm GF/F filters. Samples were treated as described above for 24-h ^14^C experiments. ^14^C-uptake rates were normalized to [Chl *a*] and light-response curves were fit to the model of Webb et al. (1974) to derive the maximum, light-saturated rate $P_{\hbox{max} }^{{Chl\,{\text{a}}}}$ (mol C (mol Chl *a*)^−1^ s^−1^), the light-depended increase in ^14^C-uptake before light saturation *α* ^RCII^ (mol C (mol Chl *a*)^−1^ s^−1^ [µmol photons m^−2^ s^−1^]^−1^), and the light saturation parameter *E*_k_ (µmol photons m^−2^ s^−1^).

Differences in the spectral distribution of the light being available for phytoplankton during the incubations, in the photosynthetron and in the FRRF instrument could potentially affect our results. Therefore, a spectral correction factor was derived and applied to correct values of σ_PSII_ (and consequently ETR_RCII_) as well as short-term ^14^C-uptake rates (Schuback et al. 2016). The spectral distribution in all three was measured using a micro-spectrometer equipped with a fiber-optic probe (STS-VIS, Ocean Optics). Furthermore, samples for phytoplankton light absorption spectra were taken from each bottle at dilution and final sampling days, and analyzed using the quantitative filter pad technique (Mitchell et al. 2002) as described in Schuback et al. (2017). Spectrally corrected rates of 2-h ^14^C-uptake and ETR_RCII_ were used to derive the conversion factor *Κ*/*n*_PSII_ (mol e^−^ mol C^−1^(mol Chl *a*) mol RCII^−1^) at light limitation (*α* ^RCII^/*α* ^Chl *a*^) and light saturation ($P_{ \hbox{max} }^{\text{RCII}}$ / $P_{\hbox{max} }^{{Chl\,{\text{a}}}}$), as described in more detail in Schuback et al. (2015, 2016).

Non-photochemical quenching (NPQ) for each light level was estimated as the normalized Stern–Volmer quenching coefficient, ${\text{NPQ}}_{\text{NSV}} = \left( {F^{\prime}_{\text{m}} /F^{\prime}_{\text{v}} } \right) - 1 = F^{\prime}_{\text{m}} /F^{\prime}_{\text{v}}$ (McKew et al. 2013). NPQ_NSV_ represents the ratio of total non-photochemical energy dissipation in the light-regulated state to the rate constant of photochemistry.

### Statistics

All data are presented as the mean of three replicates with ± one standard deviation, except for the data from the light-response curves where replicate measurements were combined before curve fitting. To test for significant differences between the treatments, two-way analyses of variance (ANOVA) with additional Kolmogorov–Smirnov normality and Tukey post hoc tests were performed. The significance level was set to 0.05. Statistical analyses were performed with the program SigmaPlot (version 12.5, SysStat Software Inc). For the light-response curves, significant differences were defined as instances where the 0.95 confidence interval of derived fit parameters did not overlap.

## Results

### Environmental conditions during sampling of the phytoplankton assemblage

The sampling location was characterized by a particularly shallow mixed layer depth (~4 m) resulting from strong haline stratification, and by nutrient depletion in surface waters. A deep Chl *a* maximum (5.4 µg L^−1^) was situated between 20 and 35 m depth (Online Resource 1, 3). Sampling was conducted just below this Chl *a* maximum corresponding to 40–45 m depth, where water temperature was −1.6 °C, salinity was 32.72, and seawater pCO_2_ was 374 µatm. PAR at this depth corresponds to 1–2% of incident irradiance (1% *I*_0_ was at 47 m). In situ macronutrient concentrations in the sampled seawater were 7.8 µmol L^−1^ NO_3_^−^, 1.1 µmol L^−1^ PO_4_^3−^, and 13.1 µmol L^−1^ Si(OH)_4_ (Online Resource 3). Chl *a* concentrations were 0.58 µg L^−1^, with 55% of the total Chl *a* in the >5-µm size fraction (Table 1). The initial phytoplankton assemblage was composed of a mix of flagellates (including *Phaeocystis* sp.), picoeukaryotes (primarily *Micromonas pusilla*), and diatoms (Table 1). Diatoms contributed to a large proportion of the assemblage, with *Chaetoceros* spp. as the most abundant genus, and relatively high abundances of *Fragilariopsis* sp. and *Thalassiosira* sp.. Average incident PAR was 415 µmol photons m^−2^ s^−1^ on the day before sampling for the experiment, with a noon maximum of 1250 µmol photons m^−2^ s^−1^ (Online Resource 4).

**Table 1:** Composition of phytoplankton assemblages at the start, immediately before the dilution and at the end of the experiment as characterized by the proportion of total Chl *a* >5-µm size fraction, POC-normalized picoplankton counts, and the dominant diatom genera (*n* = 3; mean ±1 SD)

Time point	Treatment	Chl *a* > 5 µm (% of total)	Picoplankton [cells (ng POC)^−1^]	Dominant diatom genera
Initial		55	48 ± 7^a^	Diverse assemblage of diatoms, flagellates, picoeukaryotes
Dilution	LL LC	80 ± 5	307 ± 70	*Chaetoceros socialis/gelidus*, (*Thalassiosira nordenskoeldii*)
	LL HC	79 ± 6	281 ± 75	*Chaetoceros socialis/gelidus*, (*Thalassiosira nordenskoeldii*)
	HL LC	84 ± 5	269 ± 89	*Chaetoceros socialis/gelidus*, (*Thalassiosira nordenskoeldii*)
	HL HC	80 ± 5	338 ± 72	*Chaetoceros socialis/gelidus*, (*Thalassiosira nordenskoeldii*)
Final	LL LC	90 ± 3	175 ± 82	*Chaetoceros socialis/gelidus*
	LL HC	88 ± 3	129 ± 53	*Chaetoceros socialis/gelidus*
	HL LC	92 ± 3	178 ± 80	*Chaetoceros socialis/gelidus*
	HL HC	88 ± 4	168 ± 30	*Chaetoceros socialis/gelidus*

Species in brackets are second most abundant. None of the treatments showed statistically significant effects

^a^Standard deviation between technical replicates

**Fig. 1:**
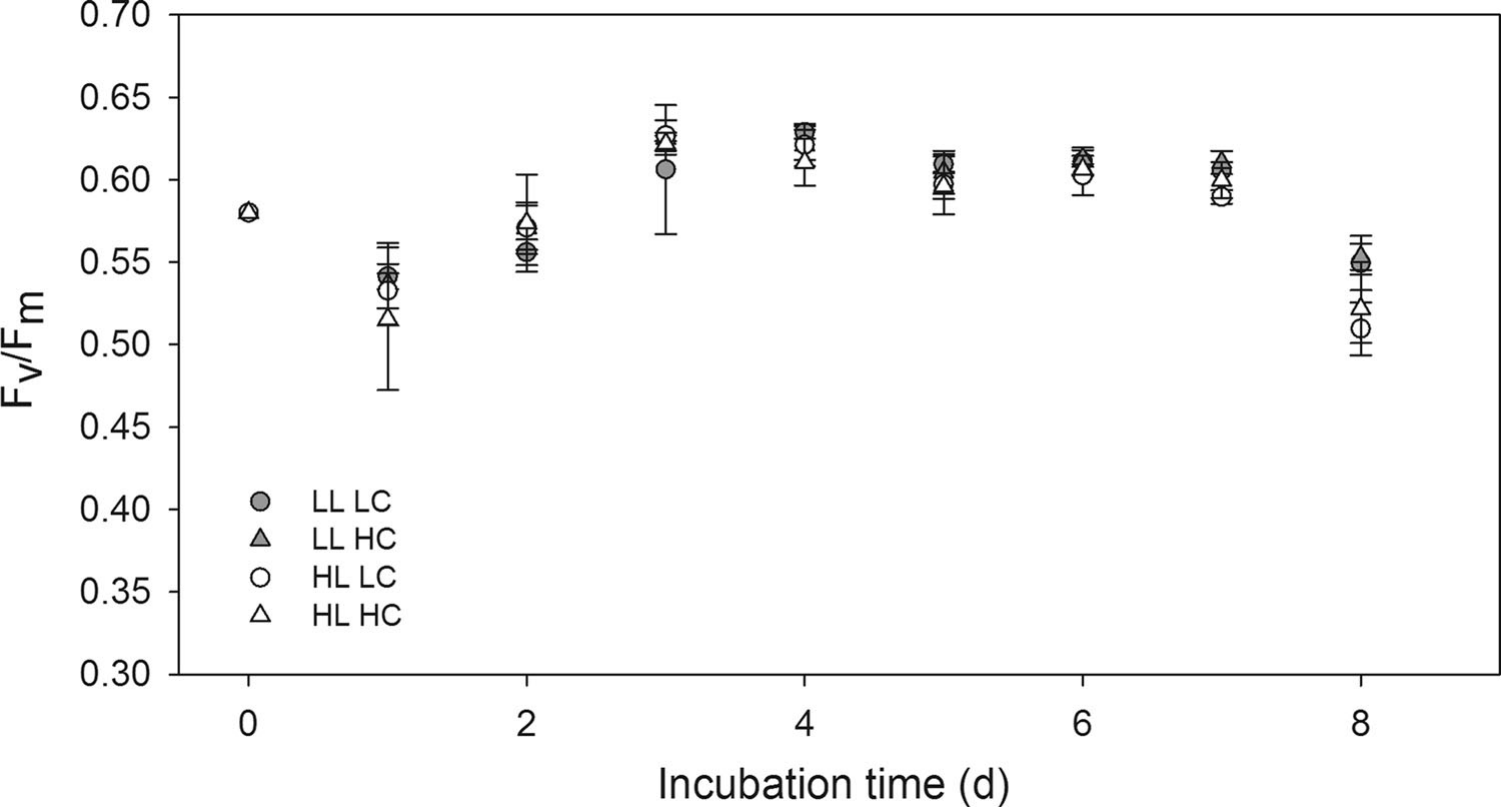
Time-course of the dark-acclimated photosynthetic quantum efficiency (*F*_v_/*F*_m_) in LL LC (*gray circles*), LL HC (*gray triangles*), HL LC (*open circles*), and HL HC (*open triangles*) treatments over the course of incubation experiment (*n* = 3 ± 1 SD). The data point at time zero represents measurements conducted with the initially sampled phytoplankton assemblage

POC-normalized NPP measured at the beginning of the experiment did not exhibit a significant light-dependent difference, with rates of 0.067 ± 0.001 and 0.072 ± 0.005 µmol C (µmol C)^−1^ d^−1^ measured in subsamples incubated in LL and HL incubators, respectively (Table 2). *F*_v_/*F*_m_ values for the initial Baffin Bay phytoplankton assemblage were close to the theoretical maximum (0.58 ± 0.01; Fig. 1), suggesting that the assemblage was not experiencing significant physiological stress prior to our incubation experiments.

**Table 2:** Growth and primary production of phytoplankton assemblages as assessed by biomass accumulation constants (*µ*), POC- and Chl *a*-specific net primary production (NPP), and Si uptake rates at the start, immediately prior to the dilution and at the end of the experiment (*n* = 3; mean ±1 SD)

Time point	Treatment	Net growth rate *µ* (d^−1^)	POC-specific NPP [µmol C (µmol C)^−1^ d^−1^]	Chl *a*-specific NPP [µg C (µg Chl* a*)^−1^ d^−1^]	Si uptake rates [µmol Si (µmol bSi)^−1^ d^−1^]
Initial	LL	n.a.	0.07 ± 0.01^a^	14.70 ± 0.31^a^	n.a.
	HL	n.a.	0.07 ± 0.01^a^	15.98 ± 1.19^a^	n.a.
Dilution	LL LC	1.33 ± 0.10	1.02 ± 0.10	43.56 ± 5.77	n.a.
	LL HC	1.22 ± 0.04	0.90 ± 0.03	37.60 ± 5.11	n.a.
	HL LC	1.32 ± 0.04	1.11 ± 0.24	43.16 ± 7.80	n.a.
	HL HC	1.27 ± 0.07	1.09 ± 0.17	44.56 ± 5.87	n.a.
Final	LL LC	1.10 ± 0.07	0.72 ± 0.01	37.89 ± 2.44	0.58 ± 0.02
	LL HC	1.03 ± 0.12	0.81 ± 0.14	42.16 ± 5.68	0.52 ± 0.14
	HL LC	1.06 ± 0.09	0.68 ± 0.01	38.15 ± 1.70	0.47 ± 0.09
	HL HC	1.07 ± 0.14	0.65 ± 0.12	39.04 ± 3.90	0.56 ± 0.26

None of the treatments showed statistically significant effects. n.a. indicates that data for the respective parameter and time point are not available

^a^Standard deviation between technical replicates

### Conditions during incubations

**Fig. 2:**
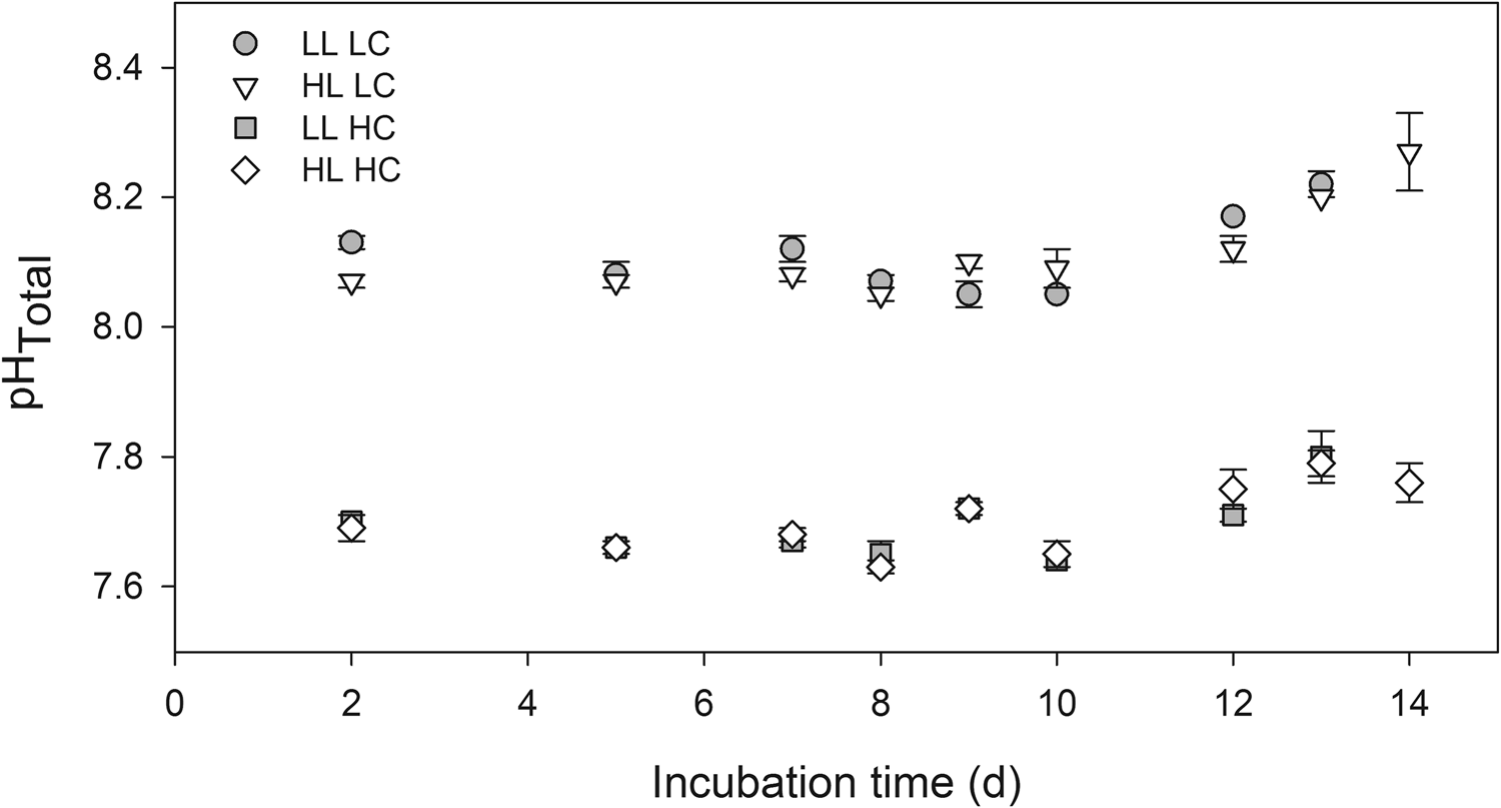
Time-course of seawater pH in incubation bottles for the LL LC (*black circles*), LL HC (*black squares*), HL LC (*gray triangles*), and HL HC (*gray diamonds*) treatments over the course of the experiment (*n* = 3 ± 1 SD). pH was measured on the total scale

Due to the experimental set-up, the phytoplankton assemblage was exposed to varying surface water temperatures and PAR levels during the 8-day experiment. Average daily incident PAR was 485 ± 125 µmol photons m^−2^ s^−1^, with maximum values of 1055 ± 105 µmol photons m^−2^ s^−1^, ranging between 905 and 1290 µmol photons m^−2^ s^−1^ (Online Resource 4). These PAR levels were reduced to 15 and 35% in LL and HL treatments, respectively. Temperatures in the flow-through incubators ranged from 4.5 to 13.0 °C and averaged 9.5 ± 1.5 °C. By comparison, in situ sea surface temperatures ranged from −1 to +7 °C, i.e., being on average 6 °C colder than in our incubators. This offset between in situ and incubator temperatures was mainly caused by warming of the seawater in the flow-through sampling system (for potential consequences see discussion chapter). Please note that, at a given time point, temperatures varied less than 0.2 °C between incubators as well as different positions therein (data not shown). Incubation temperatures generally decreased over the course of the experiment, with values of 11, 9.5, and 7 °C during the first, second, and third quarter of the experiment, respectively. Average daily pH values were 8.07 ± 0.04 and 7.66 ± 0.03 for LC and HC treatments, respectively (Fig. 2), with corresponding average pCO_2_ levels of 315 ± 30 µatm and 985 ± 55 µatm (measured before the dilution and at final sampling points; Online Resource 5). Nutrient additions at the beginning yielded concentrations of 25, 2.5, and 30 µmol L^−1^ for NO_3_^−^, PO_4_^3−^, and Si(OH)_4_, respectively. Residual nutrients before the dilution and at the final sampling were not limiting, as they remained above 15 µmol L^−1^ for NO_3_^−^ and Si(OH)_4_, and above 1.5 µmol L^−1^ for PO_4_^3−^ in all bottles.

### Responses of phytoplankton to enhanced light and OA

On the first sampling day of the experiment, the *F*_v_/*F*_m_ declined slightly from 0.58 ± 0.01 to 0.53 ± 0.04 (Fig. 1; *n* = 12). A rapid recovery to maximum levels was observed within 3 days in all treatments, irrespective of the applied irradiance. *F*_v_/*F*_m_ values did not differ significantly between treatments. Exponential growth started without a pronounced lag phase (i.e., on day 2), and the growth rates (1.2–1.3 d^−1^) did not vary significantly across treatments during the first grow-up period (i.e., before the dilution on days 1–5 of the experiment; *n* = 11; 2-way ANOVA, *F*_(1,10)_ = 0.32, *p* = 0.59 for light and *F*_(1,10)_ = 4.11, *p* = 0.08 for pCO_2_; Table 2). During the second growth phase (i.e., days 5–8, after the dilution), growth rates were about 0.2 d^−1^ lower in all treatments, again with no significant treatment effects (*n* = 12; 2-way ANOVA, *F*_(1,10)_ = 0.0001, *p* = 0.99 for light and *F*_(1,10)_ = 0.16, *p* = 0.71 for pCO_2_). The >5-µm size phytoplankton fraction, which was dominated by diatoms (Table 1), comprised about 81 ± 5% of the total Chl *a* before the dilution and 90 ± 3% during the final sampling (Table 1).

Almost none of the variables measured before the dilution and at the end of the experiment were significantly affected by any of the treatments. This was true for ratios of POC to PON (Table 3; *n* = 12; 2-way ANOVA, *F*_(1,11)_ = 0.07, *p* = 0.80 for light and *F*_(1,11)_ = 0.80, *p* = 0.40 for pCO_2_ before the dilution and *F*_(1,11)_ = 1.2, *p* = 0.30 for light and *F*_(1,11)_ = 0.17, *p* = 0.69 for pCO_2_ at the final sampling), Chl *a* (Table 3; *n* = 12; 2-way ANOVA, *F*_(1,11)_ = 2.25, *p* = 0.17 for light and *F*_(1,11)_ = 0.59, *p* = 0.47 for pCO_2_ before the dilution and *F*_(1,11)_ = 4.6, *p* = 0.06 for light and *F*_(1,11)_ = 0.19, *p* = 0.68 for pCO_2_ at the final sampling), and bSi (Table 3; *n* = 12; 2-way ANOVA, *F*_(1,11)_ = 1.78, *p* = 0.22 for light and *F*_(1,11)_ = 1.65, *p* = 0.24 for pCO_2_ before the dilution and *F*_(1,11)_ = 0.004, *p* = 0.96 for light and *F*_(1,11)_ = 3.72, *p* = 0.09 for pCO_2_ at the final sampling). Similarly, no significant differences were observed among treatments for either the relative abundance of the >5-µm size phytoplankton estimated as the Chl *a* >5-µm size fraction, which as dominated b diatoms and accounted for >80% of the total Chl *a* in all cases (Table 1; *n* = 12; 2-way ANOVA, *F*_(1,11)_ = 0.63, *p* = 0.45 for light and *F*_(1,11)_ = 0.45, *p* = 0.52 for pCO_2_ before the dilution and *F*_(1,11)_ = 0.65, *p* = 0.44 for light and *F*_(1,11)_ = 3.07, *p* = 0.12 for pCO_2_ at the final sampling), or the relative abundance of picoplankton measured by flow cytometry (Table 1; *n* = 12 before the dilution and *n* = 11 at the final sampling; 2-way ANOVA, *F*_(1,11)_ = 0.05, *p* = 0.84 for light and *F*_(1,11)_ = 0.25, *p* = 0.63 for pCO_2_ before the dilution and *F*_(1,10)_ = 0.3, *p* = 0.60 for light and *F*_(1,10)_ = 0.54, *p* = 0.49 for pCO_2_ at the final sampling). Furthermore, community growth rates (see above), Chl *a*-specific NPP (*n* = 11; 2-way ANOVA, *F*_(1,10)_ = 0.71, *p* = 0.43 for light and *F*_(1,10)_ = 0.34, *p* = 0.58 for pCO_2_ before the dilution and *F*_(1,10)_ = 0.36, *p* = 0.57 for light and *F*_(1,10)_ = 1.16, *p* = 0.32 for pCO_2_ at the final sampling), POC-specific NPP (*n* = 11; 2-way ANOVA, *F*_(1,10)_ = 1.88, *p* = 0.21 for light and *F*_(1,10)_ = 0.48, *p* = 0.51 for pCO_2_ before the dilution and *F*_(1,10)_ = 2.85, *p* = 0.14 for light and *F*_(1,10)_ = 0.27, *p* = 0.62 for pCO_2_ at the final sampling) as well as Si uptake rates (*n* = 11; 2-way ANOVA, *F*_(1,10)_ = 0.10, *p* = 0.76 for light and *F*_(1,10)_ = 0.01, *p* = 0.92 for pCO_2_ at the final sampling; not measured before the dilution) did not differ between treatments (Table 2). This was also true for most photo-physiological measurements (Table 4; *n* = 12). Here, however, interactive effects of light and pCO_2_ were observed before the dilution. After the first five days of the experiment, LL-acclimated rates of maximum electron transport (*P*_max_-ETR_RCII_ and *P*_max_-Κ_C_/n_PSII_) were significantly higher under HC than LC. In HL-acclimated cells, non-photochemical quenching capacity (NPQ_NSV_) was significantly lower under HC than LC, but this effect was not observed under LL. At the final time point, *P*_max_-ETR_RCII_ was 15% higher under HL compared to LL, irrespective of the applied pCO_2_. No other treatments effects on photo-physiology were observed.

**Table 3:** Elemental composition of phytoplankton assemblages at the start, immediately prior to the dilution, and at the end of the experiment (*n* = 3; mean ±1 SD)

Time point	Treatment	POC:PON (mol mol^−1^)	POC:Chl *a* (g g^−1^)	POC:bSi (mol mol^−1^)
Initial		7.08 ± 0.78^a^	142.5 ± 10.4^a^	6.12 ± 0.06^a^
Dilution	LL LC	5.03 ± 0.71	77.1 ± 2.5	9.33 ± 1.71
	LL HC	5.74 ± 0.80	73.9 ± 2.7	10.14 ± 1.52
	HL LC	5.19 ± 0.93	67.2 ± 2.7	7.81 ± 0.55
	HL HC	5.33 ± 0.84	66.8 ± 3.5	9.40 ± 2.34
Final	LL LC	5.02 ± 0.57	94.1 ± 0.7	5.18 ± 0.51
	LL HC	5.45 ± 0.81	95.3 ± 1.4	5.35 ± 0.55
	HL LC	5.76 ± 0.94	116.9 ± 6.0	4.73 ± 0.48
	HL HC	5.69 ± 0.73	101.8 ± 8.8	6.08 ± 1.04

None of the treatments showed statistically significant effects

^a^Standard deviation between technical replicates

**Table 4:** Photo-physiological characteristics of phytoplankton assemblages immediately before the dilution and at the end of the experiment (*n* = 3; mean ±1 SD)

Time point	Treatment	*α*-ETR_RCII_ [mol e^−^ (mol RCII)^−1^ (µmol photons)^−1^ m^2^]	*α*-*C*_fix_ [mol C (mol Chl*a*)^−1^ (µmol photons)^−1^ m^2^]	*P*_max_-ETR_RCII_ [mol e^−^ (mol RCII)^−1^ s^−1^]	*P*_max_-C_fix_ [mol C (mol Chl*a*)^−1^ s^−1^]
Dilution	LL LC	1.73 ± 0.58	0.0015 ± 0.0003	206 ± 27c	0.078 ± 0.004
	LL HC	2.21 ± 0.29	0.0012 ± 0.0002	167 ± 6	0.084 ± 0.005
	HL LC	1.55 ± 0.64	0.0014 ± 0.0002	154 ± 17	0.079 ± 0.004
	HL HC	2.24 ± 0.51	0.0011 ± 0.0002	179 ± 10	0.080 ± 0.003
Final	LL LC	2.07 ± 0.51	0.0012 ± 0.0004	191 ± 13a	0.086 ± 0.009
	LL HC	2.17 ± 0.29	0.0011 ± 0.0002	183 ± 7	0.084 ± 0.005
	HL LC	1.73 ± 0.26	0.0010 ± 0.0002	220 ± 12	0.090 ± 0.007
	HL HC	2.24 ± 0.43	0.0011 ± 0.0002	209 ± 11	0.088 ± 0.005

Time point	Treatment	*E*_k_-ETR_RCII_ [µmol quanta m^−2^ s^−1^]	*E*_k_-C_fix_ [µmol quanta m^−2^ s^−1^]	*α*-*Κ*_C_/*n*_PSII_	*P*_max_-*Κ*_C_/*n*_PSII_	NPQ_NSV_880_
Dilution	LL LC	119 ± 43	55 ± 10	1180 ± 450	2636 ± 366c	17.6 ± 7.2c
	LL HC	76 ± 10	71 ± 13	1853 ± 416	1989 ± 128	17.6 ± 9.4
	HL LC	99 ± 42	58 ± 10	1112 ± 492	1949 ± 235	20.7 ± 5.9
	HL HC	80 ± 19	71 ± 11	1987 ± 533	2246 ± 161	12.3 ± 1.9
Final	LL LC	92 ± 23	71 ± 23	1716 ± 676	2231 ± 270	12.1 ± 1.7
	LL HC	84 ± 12	73 ± 14	1906 ± 428	2190 ± 148	11.9 ± 1.3
	HL LC	127 ± 20	86 ± 21	1657 ± 455	2443 ± 240	14.3 ± 2.8
	HL HC	93 ± 19	81 ± 14	2065 ± 519	2381 ± 178	12.1 ± 0.6

Fit parameters (*α*, *E*_k_, *P*_max_) for FRRf-based ETR_RCII_ and ^14^C-uptake derived from *P* versus *E* curves as well as conversion factors between both and NPQ_NSV_ observed at 880 µmol photons m^−2^ s^−1^ as described in Schuback et al. (2015, 2016). Significant effects (*p* > 0.05) of light, or interaction of light and CO_2_ are indicated by a, or c, respectively

The centric diatom *Chaetoceros socialis*, followed by *Thalassiosira nordenskoeldii,* dominated all of the phytoplankton assemblages before the first dilution. All final assemblages were solely dominated by *C. socialis*. Please note that this species, previously identified as *C. socialis* in many studies (e.g., Booth et al. 2002; Tremblay and Gagnon 2009; Martin et al. 2010), has recently been described as a new species, i.e., *C. gelidus,* being mainly found in polar waters (Chamnansinp et al. 2013, Blais et al. 2017). For the sake of comparability with older studies, we refer to both the old and the new name in the following. No treatment effects were observed for any of the indicators of phytoplankton community structure, i.e., Chl *a* size fractions, picoeukaryote contributions, and taxonomic composition via light microscopy (Table 1). Microzooplankton grazers such as ciliates were not observed in significant abundance.

## Discussion

The aim of this study was to improve our understanding of the combined effects of enhanced light and ocean acidification on Arctic phytoplankton. We chose experimental conditions that would mimic the effects of upwelling high nutrient waters into the surface mixed layer. The phytoplankton assemblages in our experiment, all being dominated by *C. socialis/gelidus*, acclimated quickly to the different combinations of light and pCO_2_. Contrary to our expectations, neither light intensity nor pCO_2_ levels affected the eco-physiological parameters investigated. The high resistance of phytoplankton assemblages to the experimental conditions will be discussed both in the context of potential mechanisms that could explain the observed non-responsiveness, e.g., functional redundancy, as well as the implications for primary production in the future Arctic Ocean.

### Light-dependent responses were subtle and disappeared during acclimation

The phytoplankton assemblage acclimated to all experimental conditions within the first few days of the experiment (Fig. 1). The absence of measurable high-light stress after 3 days is surprising, given that assemblages originated from below the DCM at about 1–2% of incident irradiance and that weather conditions did not change significantly over the course of the experiment (Online Resource 4). Furthermore, assemblages from Davis Strait (69°N) exhibited pronounced high-light stress in a similar experiment set-up (Hoppe Clara et al. 2017). Large changes in irradiance, however, also occur during upwelling events, which play an important role in triggering autumn blooms in ice-free Arctic areas (Ardyna et al. 2014). As discussed below, Arctic phytoplankton assemblages can therefore be expected to possess mechanisms that allow them to thrive under sudden high-light conditions (Platt et al. 1982; Gosselin et al. 1990; Campbell et al. 2015; Schuback et al. 2017).

The lack of differential responses to the two applied light levels (15 and 35% of incident irradiances) is even more surprising than the fast recovery from initial high-light stress. It seems, however, plausible to assume high photo-physiological plasticity for several reasons. Firstly, high-latitude ocean waters are generally characterized by high variability in environmental conditions such as irradiances (Carmack and Wassmann 2006; Wassmann & Reigstad 2011; Tremblay et al. 2015). Furthermore, the assemblage was dominated by diatoms, which are known to possess highly efficient photo-protective machinery (Lavaud et al. 2004). Our FRRF measurements indeed revealed a high capacity for energy dissipation via NPQ (Table 4), even when compared to relatively high values previously measured in diatom-dominated Arctic phytoplankton assemblages as well as individual diatom and haptophyte strains (McKew et al. 2013; Hoppe et al. 2015; Schuback et al. 2017). Moreover, relatively high temperatures occurring in the incubators may have helped to prevent high-light stress by increasing the turnover rate of carbon fixation relative to electron transport, thereby allowing the Calvin cycle to be a more efficient sink for light energy (Mock and Hoch 2005; Goldman et al. 2015). Light effects were, however, also absent during the final sampling, i.e., under the 4.5 °C colder temperatures compared to the measurement before the first dilution (Online Resource 1). Thus, high incubation temperatures cannot be regarded as the only explanation for the lack of photo-inhibitory responses.

In addition to the lack of CO_2_ and light effects on eco-physiology, the species composition of the phytoplankton assemblages did not show appreciable responses to our experimental treatments either. All treatment bottles were dominated by the diatom *C. socialis/gelidus*, which is a potentially cosmopolitan species that regularly dominates blooms in Subarctic and Arctic waters (Hasle and Syvertsen 1997; Booth et al. 2002; Degerlund and Eilertsen 2009). The widespread distribution of this species suggests that it exhibits a particularly wide tolerance towards different environmental conditions, i.e., a high plasticity in response towards changes in light and other drivers. Our incubation results confirm this interpretation.

### Assemblages did not respond to ocean acidification

**Fig. 3:**
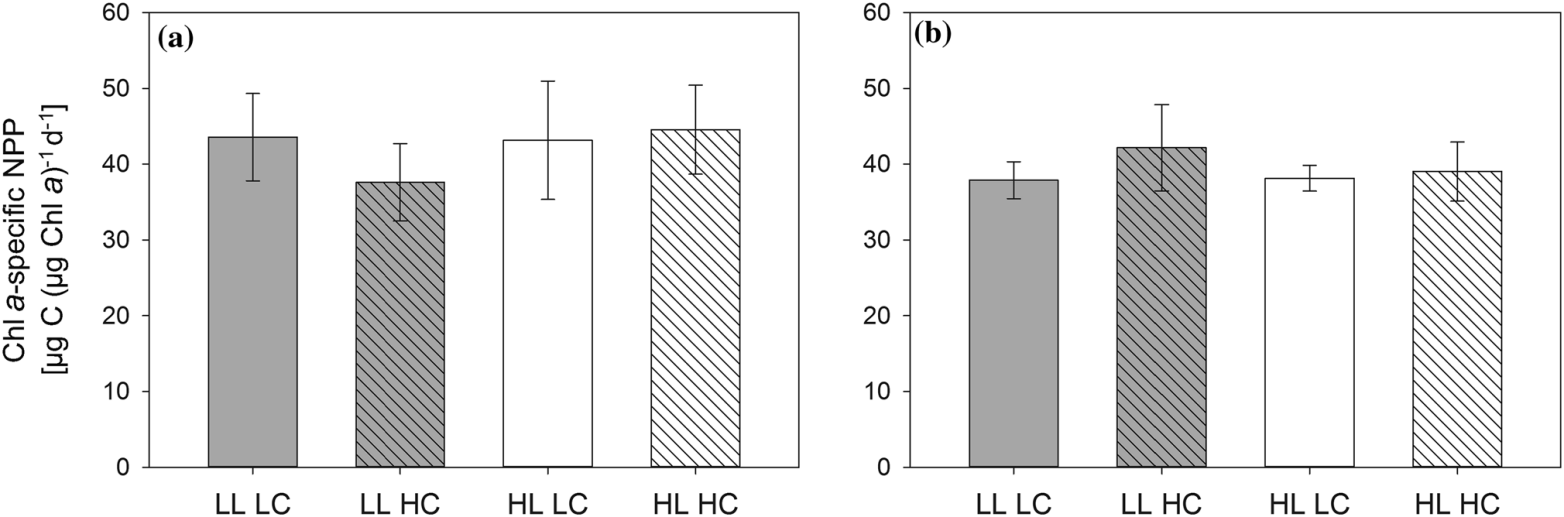
Chl *a*-specific net primary production (NPP) measured in the respective LL (*gray bars*) and HL (*open bars*) incubators at LC (*non-hatched bars*) and HC (*hatched bars*) over 24 h immediately prior to the dilution (**a**), and at the final sampling (**b**) of the experiment (*n* = 3 ± 1 SD). None of the treatments show statistically significant effects

Similar to the lack of substantial responses to high light, elevated pCO_2_ levels did not induce significant eco-physiological responses in the investigated phytoplankton assemblages. OA is studied widely because of its multiple effects on the physiology, ecology, and biogeochemistry of marine ecosystems (Pörtner et al. 2014). While some studies show a high potential for a stimulation of high-latitude NPP under elevated pCO_2_ (e.g., Tortell et al. 2008; Hoppe et al. 2013; Holding et al. 2015), other studies have demonstrated little to no stimulation of growth of phytoplankton assemblages in the range of pCO_2_ levels expected until the end of the century (Coello-Camba et al. 2014; Thoisen et al. 2015). Furthermore, whereas previous studies have demonstrated interactions between OA and high light, causing amplified high-light stress under short-term exposure (Gao et al. 2012b; Hoppe et al. 2015), we did not observe such effects. In fact, prior to the dilution, we even observed decreasing electron transport and enhanced energy conversion efficiency (*P*_max_-ETR_RCII_ and *P*_max_-*Κ*_C_/*n*_PSII_; Table 4) with increasing pCO_2_ under LL, hinting towards potentially beneficial OA effects under low irradiances (Rokitta and Rost 2012). These effects, however, did not translate into changes in primary production and furthermore disappeared over time (Fig. 3; Table 4). It remains to be understood which physiological mechanisms underlie such high capacity for photo-acclimation.

Due to the importance of biogenic silica for the efficiency of carbon export to depth (Brzezinski et al. 1998; Assmy et al. 2013), interest on the effects of OA on silicification of diatoms has been increasing. Laboratory studies indicate a reduction of biogenic silica content with increasing pCO_2_ levels (Milligan et al. 2004; Hoppe et al. 2015). To our knowledge, our study is the first to apply the ^32^Si uptake technique (Brzezinski and Phillips, 1997) to assess pCO_2_ effects on natural phytoplankton assemblages. Our results showed that OA had no effect on either Si uptake (Table 2) or bSi:C ratios (Table 3). Since the assemblages in all incubation bottles were dominated by the same species, this non-responsiveness seems to be of physiological origin. It could, however, also be caused by shifts between differentially silicified *Chaetoceros* strains compensating for potential physiological OA effects (Collins et al. 2014; Wolf et al. 2017).

In all treatments, the assemblages were dominated by *C. socialis* (or *C. gelidus* according to recent taxonomic description; Chamnansinp et al. 2013; Blais et al. 2017). *C. socialis/gelidus* was also dominating the >5 µm fraction of the initial and final assemblages of another experiment initiated with waters from within the DCM at the same time as our experiment (Hussherr et al. 2017). As *C. socialis/gelidus* has been shown to be the most abundant diatom species in the DCM throughout Canadian Arctic waters (Martin et al. 2010) and to commonly form blooms in Baffin Bay in summer (Booth et al. 2002), we conclude that its dominance can be held representative for in situ dynamics and that it is not an artifact from our experimental set-up.

In line with our results, Hussherr et al. (2017) found *C. socialis/gelidus* to dominate their experimental assemblages over the full range of the pH treatments (8.1–7.2; achieved by acid and bicarbonate addition) from an experiment initiated at the same location. Our results also agree well with previous laboratory experiments, which have shown that Antarctic *Chaetoceros* isolates show little to no stimulation by increasing pCO_2_ levels (Boelen et al. 2011; Trimborn et al. 2013; Hoppe et al. 2015). Nonetheless, this genus dominated OA treatments in several experiments with natural assemblages from the Southern Ocean, suggesting it to be a ‘potential winner of OA’ (Tortell et al. 2008; Feng et al. 2010; Boelen et al. 2011; Trimborn et al. 2013). The success of this genus seems to be at least partially due to more detrimental effects on other species rather than strongly beneficial effects on *Chaetoceros* spp. itself (Trimborn et al. 2013; Hoppe et al. 2015). We thus hypothesize that the unexpectedly high resistance of phytoplankton assemblages to CO_2_ variability on all investigated levels (ranging from photo-physiology to species composition) is caused by a high level of physiological plasticity of *C. socialis/gelidus* in the seeding population, potentially together with high intra-specific diversity causing selection between strains with different environmental optima (Schaum et al. 2012; Wolf et al. 2017).

Our results from this *C. socialis/gelidus*-dominated assemblage suggest that Arctic phytoplankon can have the capacity to buffer the effects of changing CO_2_ concentrations and pH on various levels (stoichiometry, photo-physiology, productivity, species composition). Please note that other studies observed negative effects of OA on biomass build-up only at pH levels below 7.6 (Thoisen et al. 2015; Hussherr et al. 2017), i.e., more extreme than the predicted changes for the next century. The environmental conditions prevailing in the Arctic could promote high physiological plasticity towards varying pCO_2_ levels. The Arctic marine carbonate system is indeed characterized by naturally high pCO_2_ variability, caused by the higher CO_2_ solubility under low seawater temperatures, the lower buffering capacity due to the low alkalinity, and the effects of intense seasonal primary production in some regions (Wassmann and Reigstad 2011; Shadwick et al. 2013; Thoisen et al. 2015; Burt et al. 2016). Phytoplankton species occurring in such environments thus need to be able to acclimate to a wide range of pCO_2_ levels. Furthermore, the higher fraction of inorganic carbon present as aqueous CO_2_ under low seawater temperatures may facilitate carbon acquisition, as recently shown for Antarctic phytoplankton assemblages (Kranz et al. 2015). This could reduce the beneficial effects of increased concentrations of dissolved CO_2_ under OA (Rost et al. 2008). Another possible reason for the lack of pCO_2_ effects is the relatively high incubation temperature. High temperatures have been shown to shift phytoplankton pCO_2_ optima to higher levels and could thus dampen OA effects on phytoplankton assemblages (Sett et al. 2014; Holding et al. 2015; Wolf et al. 2017). Although the incubation temperatures were higher than in situ, we argue that our experimental set-up nonetheless reflects a realistic scenario for the current, and certainly the future Arctic Ocean. The incubation temperatures at the final sampling (6.3 °C) were within the range of naturally occurring temperatures in Arctic outflow shelf regions (Beszczynska-Möller et al. 2012; Straneo and Heimbach 2013). Furthermore, such temperatures can be expected to become more common in the future (AMAP 2013; Pörtner et al. 2014). Thus, the here-observed pattern seems to be a realistic representation for Arctic phytoplankton assemblages.

### Implications for primary production in the future Arctic Ocean

Our results indicate that the resistance of Arctic phytoplankton to climate change effects may be high. Changes in light availability due to sea–ice decline and snow dynamics, as well as ongoing ocean acidification may exert a lesser effect on Arctic primary production than previously thought (see also Hussherr et al. 2017). It has been argued that sea–ice retreat and enhanced stratification will increase Arctic primary production through enhanced light availability (Arrigo and van Dijken 2011). There is, however, increasing evidence that nutrient inventories, rather than irradiance levels, set the upper limit of annual Arctic primary production (Wassmann and Reigstad 2011; Tremblay et al. 2015). In addition, our results may indicate that increased irradiances per se, with or without OA, do not enhance net primary production in Arctic phytoplankton assemblages. Nonetheless, earlier ice melt will most likely change bloom phenology and timing with potentially large implications for higher trophic levels (Wassmann and Reigstad 2011).

Our experimental set-up was chosen to simulate a wind-driven upwelling event in the stratified summer situation, where sub-surface waters are transported into the surface mixed layer, transporting phytoplankton, and nutrients into higher irradiances. Recent work has suggested that such events may become increasingly common as a result of enhanced winds and more exposed sea surface areas in autumn (Ardyna et al. 2014). Additional work is needed to elucidate whether the here-described late-summer responses can also be observed in autumn, and whether such upwelling events may occur more frequently under future summer situations. In the case of nutrient-limited surface assemblages, previous studies suggest responses similar to those observed here (Coello-Camba et al. 2014; Holding et al. 2015). A different and more diverse community structure of surface blooms in spring, however, may respond differently to the applied conditions. As incident irradiances are higher during spring, and gradients are most pronounced in the upper part of the water column, one would not expect these spring assemblages to be more susceptible to high-light stress than the ones investigated here. However, additional studies are needed to understand the degree to which observed responses depend on the experimental set-up used here, as well as on other environmental drivers such as temperature or nutrient availability. Our study is the first to indicate that plastic responses of an *Chaetoceros*-dominated assemblage can lead to resistance towards OA. Yet, the underlying mechanisms generating this resistance need to be understood.

## Electronic supplementary material

All data are archived under https://doi.org/10.1594/PANGAEA.878255.﻿ Below is the link to the electronic supplementary material.

Supplementary material 1 (PDF 540 kb)
