# Supplementary material for: Resistance of Arctic phytoplankton to ocean acidification and enhanced irradiance
Source: Polar Biol. 2017 Aug 9;41(3):399–413. doi: 10.1007/s00300-017-2186-0 (PMC6952045; doi:10.1007/s00300-017-2186-0)
Supplement: Supplementary file 1 — Supplementary material 1 (PDF 540 kb) [file 300_2017_2186_MOESM1_ESM.pdf]

Electronic Supplementary Material to Hoppe et al. (2017) in Polar Biology

Online Resource 1: CTD profiles from initial sampling location in Baffin Bay (71° 24.327' N, 68° 36.057' W) showing temperature [°C] (solid black line), σ_T [kg m^3] (solid grey line), fluorescence-based Chl *a* concentrations [µg L^{-1}] (dashed grey line) and nitrate concentrations [µmol L^{-1}] (dotted black line).

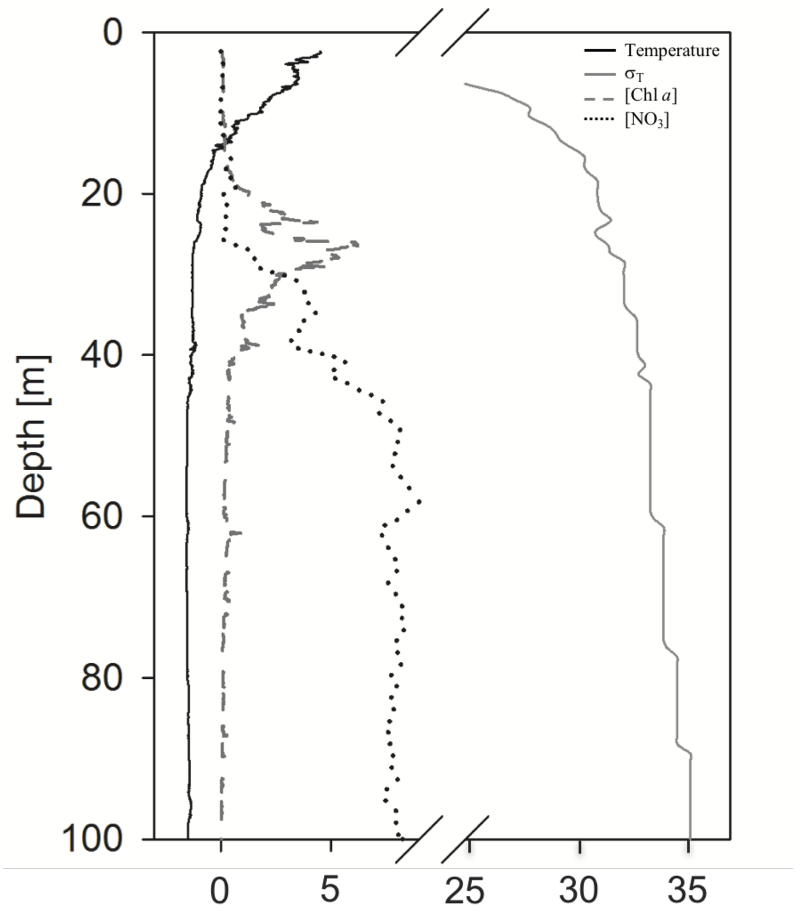

Online Resource 2: Development of dark-acclimated maximum yield estimated via FRRf in all incubation bottles over the course of the experiment. Please note that incubations were diluted on day 5 after sampling.

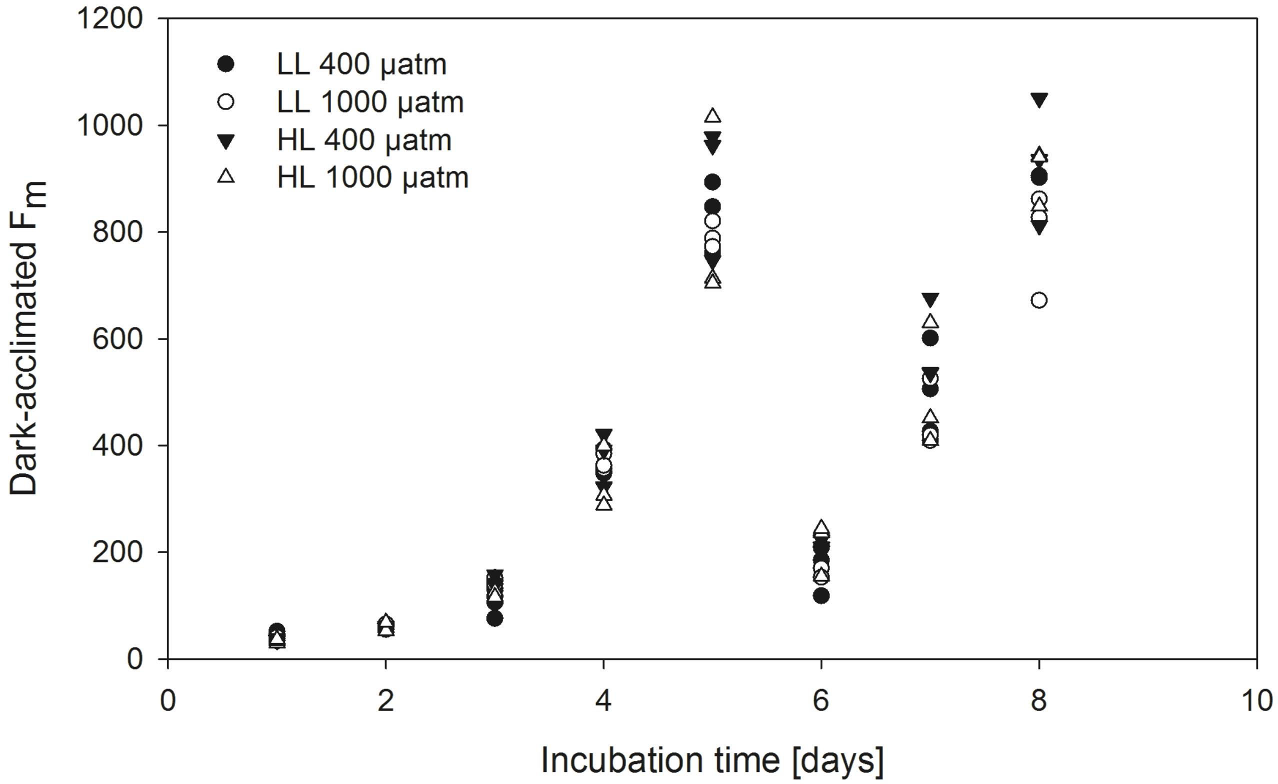

Online Resource 3: Overview of environmental conditions during sampling for the experiment.

In situ conditions	
Date of initial sampling	6-Aug-2015
Latitude	71° 24.327' N
Longitude	68° 36.057' W
Bathymetric depth [m]	1272
Mixed layer depth [m]	4
Depth of Chl*a* max [m]	20-35
Sampling depth [m]	40-45
In situ Temperature [°C]	-1.6
Salinity	32.72
Initial Chl*a* [µg L^{-1}]	0.58
Initial NO_3 [µmol L^{-1}]	7.83 ± 0.01
Initial PO_4 [µmol L^{-1}]	1.114 ± 0.002
Initial $Si(OH)_4$ [µmol L^{-1}]	13.13 ± 0.02

Online Resource 4: Incident irradiance on the day before initiation (A) as well as during experiment, both before (B) and after the dilution (C). Please note that these PAR levels were reduced to 15% and 35% in LL and HL treatments, respectively.

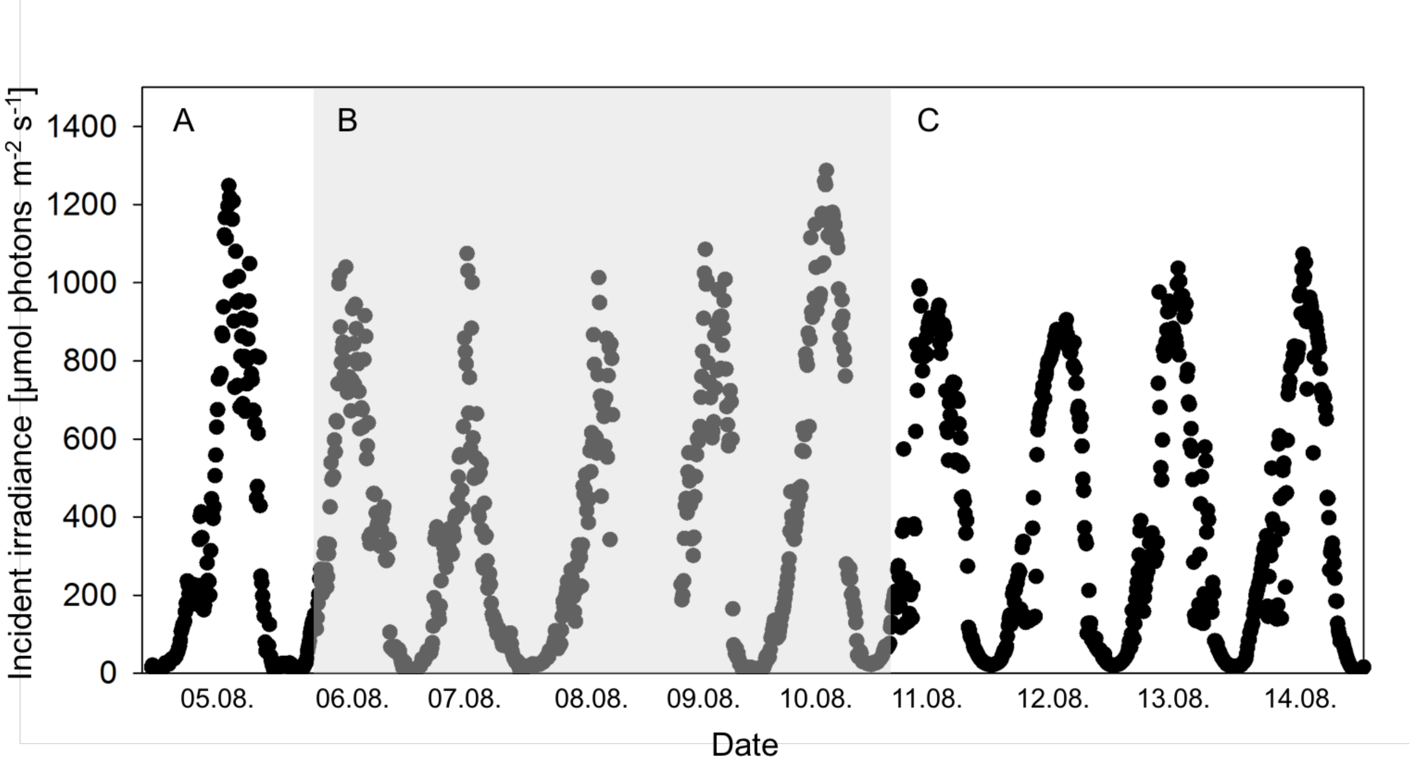

Online Resource 5: Parameters of the seawater carbonate chemistry as sampled at the beginning (*n*=1), during the dilution (*n*=3) and the end of the experiment (*n*=3; mean ±1 s.d.). pCO_2 was calculated from total alkalinity (TA) and dissolved inorganic carbon (DIC) using CO2SYS (Pierrot et al. 2006).

Timepoint	Treatment	Temperature [°C]		TA [µmol kg^{-1}]		DIC [µmol kg^{-1}]		pH_{total}		pCO_2 [µatm]	
initial		-1.6		2254		2150		8.05		374	
dilution	LL LC	10.7	± 0.5	2291	± 2	2075	± 7	8.12	± 0.02	319	± 14
	HL LC	10.7	± 0.5	2287	± 4	2089	± 8	8.09	± 0.01	353	± 11
	LL HC	10.7	± 0.5	2286	± 11	2236	± 10	7.68	± 0.03	993	± 78
	HL HC	10.7	± 0.5	2283	± 6	2239	± 4	7.66	± 0.01	1046	± 24
final	LL LC	6.3	± 0.3	2308	± 6	2103	± 3	8.17	± 0.02	282	± 14
	HL LC	6.3	± 0.3	2303	± 9	2108	± 8	8.15	± 0.03	301	± 25
	LL HC	6.3	± 0.3	2308	± 2	2269	± 6	7.71	± 0.01	920	± 29
	HL HC	6.3	± 0.3	2293	± 14	2262	± 9	7.68	± 0.02	973	± 41
